# Thyroid Function and Obesity

**DOI:** 10.4274/Jcrpe.856

**Published:** 2013-03-01

**Authors:** Silvia Longhi, Giorgio Radetti

**Affiliations:** 1 Regional Hospital of Bolzano, Department of Pediatrics, Bolzano, Italy

**Keywords:** Thyroid, obesity, ultrasound

## Abstract

Nowadays, childhood obesity is one of the biggest health emergencies in the developed countries. Obesity leads to multiple metabolic alterations which increase the risk of developing diabetes and cardiovascular diseases. Thyroid function has been often described as altered in obese children, however, it is not clear whether the altered thyroid function is the cause or the consequence of fat excess. On the other hand, thyroid structure seems also to be affected. Nevertheless, both functional and structural alterations seem to improve after weight loss and therefore no treatment is needed.

**Conflict of interest:**None declared.

## INTRODUCTION

Nowadays, childhood obesity represents one of the biggest health emergencies in developed countries; in the last 20 years, a 45% increment in the incidence of childhood obesity was observed in the USA (1). In Italy, approximately 21.2% of the subjects in the age range 0-18 years are overweight (2). Obesity frequently persists beyond childhood, and it has been estimated that about 80% of obese children become obese adults (3).

Obesity leads to multiple metabolic disorders that increase the mortality and morbidity risk in adulthood (4,5,6) and contributes in a significant way to the pathogenesis of diabetes mellitus and cardiovascular disease. Actually, many of the complications associated with obesity already exist during childhood and are specifically related to the presence of an insulin resistance (7). An early screening intended to diagnose the metabolic syndrome already in childhood would be very important, but unfortunately, criteria for this diagnosis have not been well established for children. Currently, different classifications are proposed in order to meet this diagnosis (8,9,10), but it seems difficult to establish a uniform classification system applicable universally, since many features of obesity change by ethnic group and also with the age of the patient (11). 

**Thyroid Function and Obesity **

Long before the definition of the metabolic syndrome, alterations in thyroid function were reported in obese patients. Body composition and thyroid hormones appear to be closely related since the latter is known to be involved in the regulation of basal metabolism and thermogenesis, playing an important role in lipid and glucose metabolism, food intake and fat oxidation (12,13). In agreement with this knowledge, it is well known that hypothyroidism causes a weight increase together with a decrease in basal metabolic rate and thermogenesis (14). Moreover, it has also been reported that there is an inverse correlation between free thyroxine (fT4) values and body mass index (BMI), even when fT4 values remain in the normal range (15). Lately, it has also been suggested that abnormalities in thyroid function may be secondary to weight excess. These changes, however, would still be functional, as suggested by their normalization after weight loss (16,17,18). 

**Figure 1:**
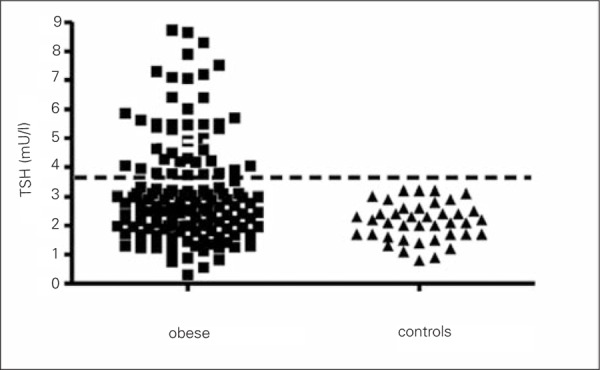
Thyroid-stimulating hormone (TSH) serum levels in a groupof 143 obese children and in normal-weight children

In various studies on adult obese individuals, thyroid hormone and thyroid-stimulating hormone (TSH) concentrations have been described as normal, elevated or reduced, compared to a control group (16,19,20,21,22,23,24). In obese children, the most common abnormality is hyperthyrotropinemia (17,18,25,26), as can be seen in Figure 1 which depicts the TSH values in a group of children affected by simple obesity and in a control group. 

The causes underlying these alterations are not known although several theories have been proposed. These include an increased deiodinase activity, as suggested by the increase in total triiodothyronine (T3) and free T3 (fT3) reported in some subjects (26). The reported high conversion rate of T4 to T3 in obese patients has been also interpreted as a defense mechanism, capable of counteracting the accumulation of fat by increasing the energy expenditure (27,28), basal metabolic rate and the total energy expenditure, being in fact positively related to the levels of total T3 and fT3. Leptin, a hormone produced by adipocytes (29), also alters the activity of deiodinases, thus promoting the conversion of T4 to T3 (30). 

Another mechanism claimed to explain the high values of T3 and fT3 has been related to the fact that the expressions of both TSH and thyroid hormones are reduced in adipocytes of obese subjects as compared to individuals of normal weight. This would prompt a decreased tissue responsiveness to circulating thyroid hormones and would also explain the consequent increased compensatory secretion of TSH and fT3 in an attempt to force the state of peripheral resistance (31). 

Another potential cause of increased blood concentration of TSH may be the high levels of leptin, found in obese subjects (25). The main action of leptin is to report centrally the amount of fat, leading to a decrease in appetite and food intake (32). In case of obesity, increased leptin is considered as an evidence of “leptin resistance” (32). In addition to this action, leptin has also been shown to stimulate centrally the transcription of pro thyrotropin-releasing hormone (TRH) and consequently also that of TRH and TSH (26). This increase in TSH, and therefore in T3, could be interpreted as a defense mechanism of the body against weight gain. In agreement with this interpretation, we observe the opposite in anorexia nervosa, in which low levels of fT3 and TSH (27) are interpreted as signs of a physiological adaptation to reduce metabolic energy expenditure (25). Moreover, TSH receptors are also localized in adipose tissue (33) and thus TSH may directly stimulate the production of leptin by adipocytes (34). Another explanation might be the impaired feedback due to a lowered number of T3 receptors in the hypothalamus (35). 

A further explanation could be the inflammatory state that characterizes obesity. It is well recognized that in obesity, the adipose tissue secretes a distinct quantity of inflammatory cytokines, and some of these, such as tumor necrosis factor-a, (TNF-a) interleukin-1 (IL-1) and interleukin-6 (IL-6), escape into the general circulation provoking systemic symptoms (36). The secretion of these cytokines, which have been proven to inhibit sodium/iodide symporter (NIS) mRNA expression and iodide uptake activity in Fisher rat thyroid cell line (FRTL-5) and human thyroid cells (37,38,39), might therefore explain the compensatory raised TSH level in obese individuals. This would also explain the tissue resistance to TSH and additionally its reversibility after weight loss.

**Normalization of Thyroid Function After Weight Loss **

**Figure 2 f2:**
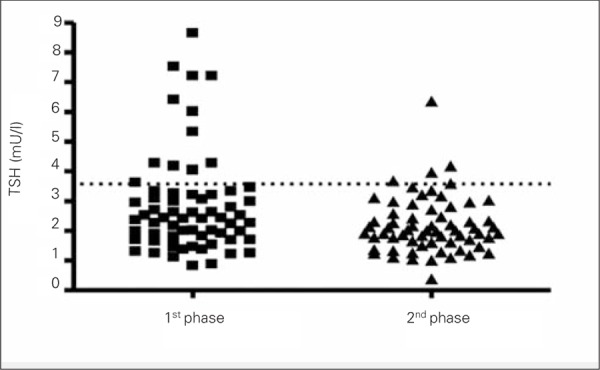
Thyroid-stimulating hormone (TSH) serum values before (1stphase) and after weight loss (2nd phase) in a group of 72 obese children

Weight loss induces a significant decrease in serum fT3 and TSH levels (16,17,18) ([Fig f2]Figure 2). The decrease in thyroid hormones, which consequently also leads to a decrease in energy expenditure, would explain the difficulty to maintain the weight loss (40). Apart from weight reduction, it seems that even simple changes of lifestyle, characterized by increased physical activity and improvement in body composition without concomitant changes of BMI, also lead to a decrease of TSH and fT3 (41). An explanation for these findings might be that weight loss or a modification in body composition reduces the state of inflammation which is present in these patients, leading to a decrease in the secretion of cytokines and therefore to a decreased inhibition on NIS and explaining therefore the improved function of the thyroid tissue (42). 

**Thyroid Ultrasound Features in Obese Patients **

**Figure 3 f3:**
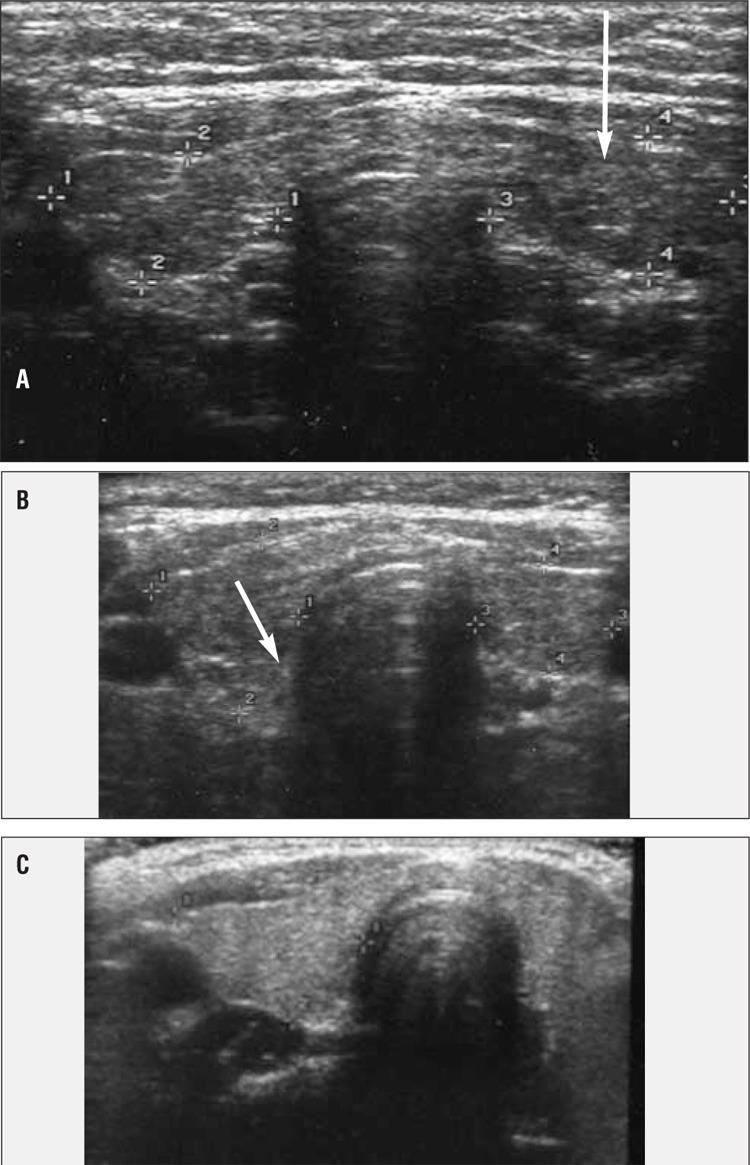
Thyroid ultrasound images in a subject with Hashimotothyroiditis (A), in an obese child (B) and in a normal subject (C)

**Figure 4 f4:**
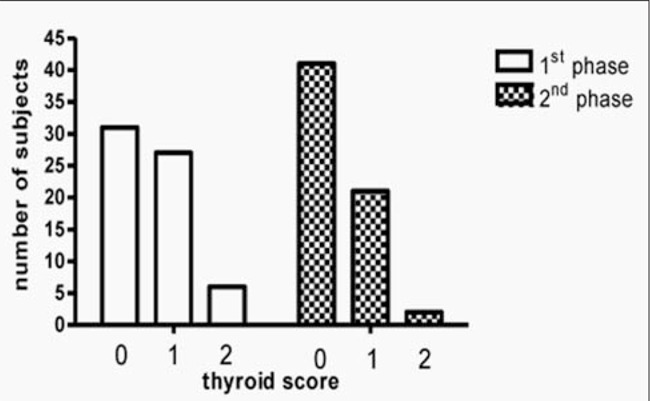
Thyroid score before (1^st^ phase) and after weight loss (2^nd^ phase)

An association between obesity and increased thyroid volume has been reported in some studies, but recently, it has also been shown that obese pediatric patients frequently have an ultrasound pattern of the thyroid which is highly suggestive of Hashimoto’s thyroiditis. These findings are not associated, however, with production of thyroid autoantibodies ([Fig f3]Figure 3) (43). Similar observations have been reported also in an adult population of obese patients (44). The cause of these findings remains unknown. A thyroid fine-needle biopsy did not show any lymphocytic infiltrate, thus excluding a typical Hashimoto’s thyroiditis. A possible but not proven cause might be, again, the low-grade inflammatory state that characterizes obesity (45). The adipose tissue in obese patients secretes inflammatory cytokines and some of these (such as TNF-α, IL-1, IL-6), entering the blood stream, can cause systemic symptoms (45). These cytokines, which contribute to increase the TSH, induce also vasodilation and increased permeability of blood vessels in the thyroid, thus causing plasma exudation and imbibition of the parenchyma; these changes may be responsible for the ultrasound findings. If this interpretation is correct, this would also explain why after weight loss and changes in lifestyle, there is a recovery of the structure of the thyroid gland ([Fig f4]Figure 4) (41).

The state of fitness, in fact, and the reduction of the inflammatory state that follows the weight loss lead probably to the regression of parenchyma imbibition (36,41,46). On the other hand, a typical Hashimoto’s thyroiditis may be present in obese children as well, and even with a higher frequency, but in most of these cases, thyroid autoantibodies are usually present (43). 

In conclusion, obese children may show different degrees of alterations pertaining to thyroid function and therefore, caution is recommended when diagnosing Hashimoto’s thyroiditis in these patients. The diagnosis should not be based just upon a pathological ultrasound, without establishing the presence of anti-thyroid antibodies. Regarding treatment, in agreement with others (18), we do not believe these children require any treatment, firstly, because the alterations found are only functional and revert to normal after weight loss or modifications in life style and secondly, because in all patients, despite a raised TSH, normal or even elevated peripheral thyroid hormone levels have been reported in all studies. This condition does not fit with the definition of subclinical hypothyroidism, in which treatment might be considered.

**Acknowledgements **

We are indebted to Dr. Lucio Parmegiani for revising and correcting the manuscript.
